# Recombinant of the Staphylococcal Bacteriophage Lysin CHAP_k_ and Its Elimination against *Streptococcus agalactiae* Biofilms

**DOI:** 10.3390/microorganisms8020216

**Published:** 2020-02-06

**Authors:** Yuxue Shan, Na Yang, Da Teng, Xiumin Wang, Ruoyu Mao, Ya Hao, Xuanxuan Ma, Huan Fan, Jianhua Wang

**Affiliations:** 1 Gene Engineering Labotory, Feed Research Institute, Chinese Academy of Agricultural Sciences, Beijing 100081, China; syx1098840130@126.com (Y.S.); nana_891230@126.com (N.Y.); tengda@caas.cn (D.T.); wangxiumin@caas.cn (X.W.); rain_mry@126.com (R.M.); haoya@caas.cn (Y.H.); 13121259099@163.com (X.M.); 2 Key Laboratory of Feed Biotechnology, Ministry of Agriculture and rural affairs, Beijing 100081, China; 3 Tianjin Animal Science and Veterinary Research Institute, Tianjin 300381, China; 4 College of Life Sciences, Tianjin Normal University, Tianjin 300387, China

**Keywords:** Lysin, CHAP_k_, expression, anti-biofilm ability, *Streptococcus agalactiae*

## Abstract

Bovine mastitis is the most important infectious disease, causing significant losses in the dairy industry, in which *Streptococcus agalactiae* is a major pathogen. In this study, lysin CHAP_k_, derived from bacteriophage K, was expressed heterogeneously, and its antimicrobial and anti-biofilm effects against *S. agalactiae* isolated from bovine mastitis were further analyzed. CHAP_k_ was expressed in *Escherichia coli* BL21 (DE3), in which the purified yield of CHAP_k_ was up to 14.6 mg/L with the purity of 95%. Time-killing kinetic curves showed that CHAP_k_ fastly killed *S. agalactiae* in TSB medium and in milk within 25 min (by 3.3 log_10_ CFU/mL and 2.4 log_10_ CFU/mL, respectively). Observation of scanning electron microscope (SEM) showed cells wrinkled and ruptured after the treatment of CHAP_k_. CHAP_k_ effectively inhibited early biofilms by 95% in 8 × MIC, and eradicated mature biofilms by 89.4% in 16 × MIC. Moreover, CHAP_k_ killed 99% bacteria in mature biofilms. Confocal laser scanning microscopy (CLSM) also demonstrated the potent antimicrobial and anti-biofilm action of CHAP_k_. It was firstly demonstrated CHAP_k_ had the characters of inhibition/elimination of *S. agalactiae* biofilms and killing the bacteria in biofilms. CHAP_k_ has the potential to develop a new antibacterial agent for mastitis treatment of *S. agalactiae* infections.

## 1. Introduction 

*Streptococcus agalactiae* of group B (GBS) is one of the important pathogens which is associated with clinical/subclinical mastitis in bovine and neonatal sepsis and meningitis in humans. Bovine mastitis is the primary health hazard, leading to severe reduction of milk production and effect of milk quality. It is responsible for significant economic losses in the dairy industry worldwide [1,2]. Infection control measures of *S. agalactiae* were performed during the 1960s to reduce the occurrence of *S. agalactiae* mastitis in several European countries. However, the prevalence of *S. agalactiae* mastitis in bovine has re-emergence in Norway, Denmark, and other countries [3,4,5]. In China, *S. agalactiae* is also a prevalent problem in bovine diagnosed with subclinical mastitis [6]. The sequence types (STs) of *S. agalactiae* in bovine mainly belong to ST67, ST103, and ST568, which harbor the virulence characteristics of biofilms formation ability, growth ability in milk and adhesion ability, and can persist indefinitely within the mammary gland [1,7]. β-lactam antibiotics (BLAs) (penicillin, ampicillin, cefalexin, and ceftiofur sodium) are officially approved and extensively used to treat *S. agalactiae* mastitis. However, clinical isolated *S. agalactiae* from cows with mastitis showed unpredicted mutations in the penicillin-binding proteins encoding (PBP) genes which lead to the high BLAs-resistant rate [8]. Eight *S. agalactiae* strains isolated from cow mastitis showed 37.5% resistant to tetracycline and were highly resistant to trimethoprim [9]. Therefore, there is an urgent need to search for novel agents against *S. agalactiae* with low possibility to develop resistance. 

Lysins are peptidoglycan hydrolase produced by bacteriophages. Among the different sources of bacteriophages, lysins show specificity on the pathogen. Compared with phages, non-biological lysins are safer and have no effect on commensal microflora [10,11]. Previous studies have demonstrated that lysins were active against streptococcus and staphylococcus pathogens, causing mucosal and systemic infections [12,13,14,15]. Lysins rapidly induce bacterial lysis and death by hydrolysing covalent bonds, which are essential for cell wall integrity and viability, and consequently develop little resistance compared with antibiotics [16,17,18]. CHAP_k_ (18.6 kDa), comprised solely of the lytic domain, is a truncated derivative of native lysin (LysK, 54 kDa) of *Staphylococcus aureus*, which belongs to cysteine-and histidine-dependent amidohydrolase/peptidase and specifically cleaves staphylococcal cell wall on the peptide bond between D-alanine and the first glycine in the pentaglycine cross bridge [19,20,21]. Previous studies have reported that CHAP_k_ exhibited strong activity against *S. aureus* and effected on the *S. aureus* biofilms [22,23,24,25]. Interestingly, researchers discovered that CHAP_k_ also had antibacterial activity against streptococcus, which may be related to the similar peptidoglycan cross-bridge of staphylococcal and streptococcal pathogens [19,26]. In this study, *CHAP_k_* gene was synthesized and expressed in *E. coli* BL21 (DE3), and the efficacy of recombinant CHAP_k_ against *S. agalactiae* biofilms was evaluated.

## 2. Materials and Methods

### 2.1. Strains, Plasmid, and Reagents

The tested strains of *Streptococcus agalactiae* CAU-FRI 1, *S. agalactiae* CAU-FRI 2, and *S. agalactiae* CAU-FRI 3 isolated from bovine mastitis were obtained from China Agricultural University, *S. agalactiae* CAU-FRI 4 isolated from tilapia was stored at our laboratory, *Streptococcus uberis* CAU-FRI 1, *S. uberis* CAU-FRI 2, *S. uberis* CAU-FRI 3, *Streptococcus dysgalactiae* CAU-FRI 1, *S. dysgalactiae* CAU-FRI 2, and *S. dysgalactiae* CAU-FRI 3 isolated from bovine mastitis were obtained from China Agricultural University, *S. agalactiae* ATCC 13813, *S. aureus* ATCC 43300, *Streptococcus epidermidis* ATCC 12228, *S. epidermidis* ATCC 35984, and *Escherichia coli* ATCC 25922 were purchased from the American Type Culture Collection; *S. aureus* CVCC 546, *Streptococcus pneumoniae* CVCC 3309, *S. pneumoniae* CVCC 2350, *Streptococcus suis* CVCC 606, *S. suis* CVCC 3928, and *Salmonella enteritidis* CVCC 3377 were purchased from the China Veterinary Culture Collection Center (Beijing, China); *S. aureus* KY, *S. aureus* KR and *S. aureus* FJ isolated from bovine with endometritis were stored at our laboratory; *Staphylococcus hyicus* NCTC 10350 was purchased from National Collection of Type Cultures; *S. pneumoniae* CGMCC 1.8747 and *Candida albicans* CGMCC 2.2411 were purchased from China General Microbiological Culture Collection Center. pET28a vector and *E. coli* BL21 (DE3) (Novagen, Beijing, China) were used for cloning and expression. DNA restriction enzymes and T4 DNA ligase were purchased from NEB (USA). The kits for plasmid extraction and DNA purification were purchased from TIANGEN (China). Other chemical reagents were analytical grade.

### 2.2. Expression and Purification of CHAP_k_

The synthesized *CHAP_k_* (The sequence was acquired from NCBI PDB:4CT3_D and synthesized by Sangon Biotech (Shanghai) Co., Ltd.) nucleotide sequence was optimized by reverse translate tool (http://bioinformatics.org//sms2/rev_trans.html) based on the preferential codon usage of *E. coli* (http://www.kazusa.or.jp/codon/) and amplified with primers of *CHAP_k_*-F: 5′-CATGCCATGGCGAAAACCCAGGCGGAAA-3′, *CHAP_k_*-R: 5′-CCGCTCGAGCTATTAGTGGTGGTGGTG-3′. The DNA fragment contained the *Xho*I and *Nco*I restriction site, and 8 × His-tag was added to the C-terminus. After confirmed by sequencing the polymerase chain reaction (PCR) fragment, the purified *CHAP_k_* fragment digested with *Xho*I and *Nco*I and ligated into pET28a vectors which digested with the same restriction enzymes to generate the pET28a-CHAP_k_ plasmid, and the plasmid was transformed into *E. coli* BL21 (DE3). The positive transforms were screened on luria broth (LB, 1% NaCl, 0.5% Yeast extract, 1% Tryptone) plates containing 50 μg/mL kanamycin and confirmed by PCR and DNA sequencing. 

The recombinant CHAP_k_ protein was expressed in *E. coli* BL21 (DE3) by IPTG induction [27]. In brief, 1% of the positive transformant cultured overnight was inoculated into LB medium containing 50 μg/mL kanamycin and incubated to OD600 nm of 0.4–0.6. CHAP_k_ expression was induced by IPTG at 1.0 mM, 37 °C for 6 h. The pellets were harvested by centrifugation at 4722 g for 5 min and analyzed by 12% SDS-PAGE. Cells were lysed by sonicated for 15 min at 0 °C, then collected precipitation and dissolved it in the binding buffer (20 mM sodium phosphate, 500 mM NaCl, 5 mM imidazole, 8 M Urea), samples were loading into Ni^2+^–Nitriloacetate (NTA) super flow resin column at a rate of 4 mL/min with elution buffer (20 mM sodium phosphate, 500 mM NaCl, 175 mM imidazole, 8 M Urea, pH 7.4). Renaturation of the CHAP_k_ by TGE buffer (50 mM Tris, 50 mM NaCl, 0.5 mM Ethylenediaminetetraacetic acid disodium salt (EDTA-Na_2_), 0.1% L-Arginine, 10% glycerol, 6 M–4 M–2 M–1 M Urea gradient dialysis until changed to distilled water) at 4 °C. To confirm the purified protein, the mixture of purified protein and matrix SA was detected by matrix-assisted laser desorption/ionization-time of flight mass spectrometry (MALDI-TOF MS) (New UltrafleXtreme, Bruker, Germany) in linear mode (at the Laboratory of Proteomics, Institute of Biophysics, Chinese Academy of Sciences). Purified protein was freeze-dried and stored in –20 °C [28].

### 2.3. Antibacterial Activity Assay

The minimal inhibitory concentrations (MICs) of CHAP_k_ were measured by the broth microdilution method. Overnight cultures were inoculated to the tryptic soy broth medium (TSB, Qingdao Hope Bio-Technology Co., Ltd.) containing 3% (*w*/*v*) BSA, cultured to the mid-log phage. The bacterial suspension was then diluted to 1 × 10^5^ CFU/mL. CHAP_k_ was two-fold diluted to final concentration with 0.625–1280 μg/mL. Then CHAP_k_ (10 μL) and bacterial suspension (90 μL) were added into 96-well plates (polystyrene, Beijing Huamei World Trade Technology Development Co., Ltd.). The plates were incubated at 37 °C for 16–18 h [29]. All assays were performed in triplicate. The MIC was defined as the lowest protein concentration at which there was no visible bacterial growth.

### 2.4. Time-Killing Kinetic Curves in TSB and Milk 

After *S. agalactiae* ATCC 13813 inoculated into TSB containing 3% BSA at 37 °C and 250 rpm for 3 h, the mid-log phase bacteria were diluted to 5 × 10^8^ CFU/mL. CHAP_k_ was mixed with bacteria suspension to the final concentration of 16 × MIC (1.72 μM) in TSB, 32 × MIC (3.44 μM) in pasteurized whole-fat milk (6% fat, 2% carbohydrate, Beijing Sanyuan food Co., Ltd.). Meanwhile, the bacteria suspension without CHAP_k_ as the blank control, and 16 × MIC and 32 × MIC of vancomycin as the positive control, respectively. Sample (100 μL) was taken at 1, 3, 5, 10, 15, 20, and 25 min for colony counting on the Tryptic Soy Agar (TSA, Qingdao Hope Bio-Technology Co., Ltd.).

### 2.5. Scanning Electron Microscope (SEM) Observation

The mid-log phase *S. agalactiae* cells were incubated with 8 × MIC CHAP_k_ for 2 h at 37 °C. After being washed with PBS for three times, *S. agalactiae* cells treated with CHAP_k_ were fixed with 2.5% glutaric dialdehyde (0.01 M PBS) at 4 °C overnight. Fixation fluid was discarded, and cells were washed with deionized water three times to removed fixation fluid for 4, 5, and 6 min, respectively. Then the samples were covered on the coverslips and various concentrations of ethanol (50%, 70%, 85%, 95%, and 100% × 3 times) were dehydrated for 15 min. Finally, the samples were dried by CO_2_, coated with platinum, and observed on a QUANTA200 SEM [30,31].

### 2.6. Ability of CHAP_k_ against Biofilms and Bacteria of S. agalactiae

#### 2.6.1. Biofilms Formation Assay

The ability of biofilms formation was evaluated by crystal violet staining, which was referenced as described by Tremblay et al. [32]. Briefly, *S. agalactiae* ATCC 13813 cells were inoculated to TSB medium containing 3% BSA, incubated until mid-log phase, and diluted to 1 × 10^8^ CFU/mL by TSB medium. A 200 μL bacterial suspension was inoculated into 96-well plates. After incubation for 24 h at 37 °C, the medium was gently rinsed by PBS for three times, excess liquid was dried in room temperature with an inverted position, and the wells were fixed with 2.5% glutaric dialdehyde for 1.5 h at 4 °C. After being washed with PBS for three times, 100 μL 0.1% crystal violet were filled into wells for dyeing and incubated for 15 min. Following removal of the crystal violet solution, the plates were washed with PBS and dried. The crystal violet was dissolved in 200 μL 95% ethanol for 30 min. The value of OD570 nm was measured, and the blank control was TSB medium with the same operation [33]. Identification by crystal violet staining: the mean OD570 of the blank control was ODc, OD was the mean value of tested strain, and the biofilm formation ability of the strain was divided into four categories: negative (-): OD < ODc; A small amount (1 +): ODc < OD ≤ 2 ODc; Medium (2 +): 2 ODc < OD ≤ 4 ODc; A large amount (3 +): OD > 4 ODc.

#### 2.6.2. Effect on Early Biofilms Formation 

Mid-log phase *S. agalactiae* ATCC 13813 cells were diluted to 1 × 10^8^ CFU/mL by TSB medium. A 180 μL bacterial suspension and a range of concentrations (0.25–8 × MIC) of CHAP_k_ (20 μL) were tested and the most effective concentration was chosen for the subsequent biofilm assays. The plates were cultured for early biofilms formation for 24 h at 37 °C, then the effect of CHAP_k_ on biofilms formation was evaluated by crystal violet staining, as described above [33]. The untreated bacteria were used as control (A). The inhibition effect of CHAP_k_ on biofilms was determined by the following equation: Biofilms (%) = [(A-A_CHAPk_)/A] × 100.

#### 2.6.3. Effect on Mature Biofilms

*S. agalactiae* ATCC 13813 cells (1 × 10^8^ CFU/mL) were cultured in TSB medium in 96-well plates at 37 °C for 24 h. Following the final concentration of 0.25–8 × MIC CHAP_k_ were mixed into the plates and cultured for another 24 h, the plates were cultured for mature biofilms formation and dyed by crystal violet, as described above [33].

#### 2.6.4. Effect on Bacteria in Early Biofilms

The mid-log phase *S. agalactiae* ATCC 13813 cells (1 × 10^8^ CFU/mL) were inoculated into in 96-well plates and cultured for 24 h at 37 °C. Plates were washed twice by PBS. Subsequently, the final concentration of 0.5–64 × MIC CHAP_k_ or vancomycin were added into plates for 2 h at 37 °C, with PBS added wells as negative control. The plates were treated with ultrasound for 5 min, and the viable bacteria were counted on the TSA plates [33].

#### 2.6.5. Effect on Bacteria in Mature Biofilms

Bacteria in mature biofilms were obtained by the following operation: the *S. agalactiae* ATCC 13813 cells (1 × 10^8^ CFU/mL) were incubated in 96-well plates (200 μL/well) for 24 h, and the planktonic bacteria were rinsed by PBS. Biofilms were incubated in 200 μL TSB containing 25 × MIC vancomycin for another 24 h at 37 °C, then the biofilm-encased bacteria were counted after the planktonic bacteria were gently removed by PBS. Subsequently, biofilms were treated with 8 × MIC of CHAP_k_ and vancomycin for 24 h at 37 °C, and the same volume of PBS was treated as control (CK), and viable bacteria in biofilms were counted by colonies counting on TSA plates [33].

#### 2.6.6. Observation of Biofilms by Confocal Laser Scanning Microscopy (CLSM)

*S. agalactiae* ATCC 13813 cells (1 × 10^8^ CFU/mL) were seeded in a confocal dish, CHAP_k_ or vancomycin were added into the dish at the final concentration of 8 × MIC and incubated for 24 h, the bacteria treated without CHAP_k_ or vancomycin were used as control. Planktonic bacteria were gently rinsed twice, and the biofilms were dyed with SYTO9 and propidium iodide (PI) (LIVE/DEAD BacLight Bacterial Viability Kit) for 15 min. After being washed with PBS, the biofilms were observed by Zeiss LSM880 confocal microscope [33].

### 2.7. Statistical Analysis

All data were analyzed by GraphPad Prism 6 and the results are presented as means ± standard deviation (SD). A *p*-value of <0.05 was considered statistically significant.

## 3. Results

### 3.1. Expression and Purification of CHAP_k_

**Figure 1:**
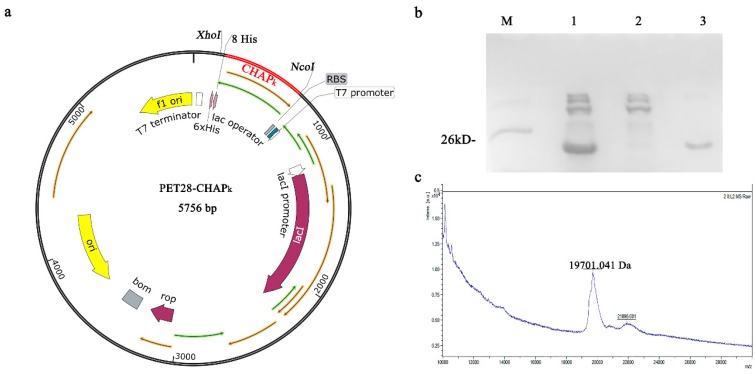
Expression and purification of CHAP_k_ in *E. coli*. (**a**) The schematic diagram of the pET28a-CHAP_k_ expression vector. (**b**) SDS-PAGE analysis of the purified CHAP_k_. M: Protein ladder, lane 1: total protein of *E.coli* BL21-pET28a-CHAP_k_, lane 2: the peak of penetration, lane 3: the peak of elution. (**c**) MALDI-TOF MS analysis of the purified CHAP_k_.

*CHAP_k_* nucleotide sequence was successfully inserted into the *Xho*I and *Nco*I sites of pET28a to construct the pET28a-CHAP_k_ plasmid after being confirmed by DNA sequencing (Figure 1a). The results of SDS-PAGE analysis showed that the CHAP_k_ with C-terminal 8 × His-tag was purified by a one-step affinity chromatography, and the target peak band was about 19 kDa (Figure 1b). The MALDI-TOF MS analysis showed that the molecular mass of CHAP_k_ is 19.701 kDa, which is consistent with its theoretical value of 19.683 kDa. The yield and purity of the CHAP_k_ were 14.6 mg/L and 95%, respectively. These results indicated that CHAP_k_ is successfully expressed in *E. coli*. 

### 3.2. Antibacterial Activity Assay

The antimicrobial activity of CHAP_k_ was determined by MICs assay (Table 1). The MIC values of CHAP_k_ against *S. agalactiae* were 0.05–0.11 μM, which were better than those of vancomycin (0.67 μM). CHAP_k_ also had the potent antimicrobial activity against other streptococcus, with MICs of 0.03–0.11 μM against *S. uberis*, 0.11–0.22 μM against *S. dysgalactiae*, 0.05–0.86 μM against *S. epidermidis*, 0.11 μM against *S. suis*, which were superior to those of vancomycin (streptococcus: 0.34–2.69 μM). For the *S. aureus* strains, the MIC values of CHAP_k_ were 0.43–3.44 μM, that of *S. hyicus* were 1.72 μM, those of *S. epidermidis* were 0.86–1.72 μM. There was almost no antimicrobial activity against Gram-negative and *C. albicans* (>6.88 μM). These results suggested that CHAP_k_ displays potent antimicrobial activity for staphylococcus and streptococcus, especially for streptococcus.

### 3.3. Time-Killing Kinetic Curves in TSB and Milk

**Figure 2:**
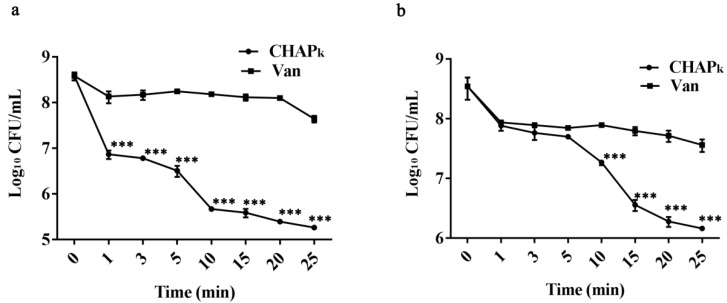
Time-killing kinetic curves in TSB and milk (**a**) Time-killing kinetic curves in TSB medium. (**b**) Time-killing kinetic curves in milk. All assays were performed in triplicate. The analyses were measured by one-way ANOVA, with Duncan’s multiple comparisons test. A probability value of < 0.05 was considered significant. (*) Indicates the significance between control and treatment groups. ***p < 0.001. The results are given as the mean ± SD (*n* = 3).

Assessment of bactericidal activity of CHAP_k_ was performed by time-killing kinetic curves. This is an important indicator of efficacy evaluation, indicating the bactericidal rate of CHAP_k_. The results showed that CHAP_k_ displayed rapid bactericidal activity. With 16 × MIC CHAP_k_ incubation, the bacteria number were reduced by 1.7 log_10_ CFU/mL in 1 min, which was four times higher than that of vancomycin (0.4 log_10_ CFU/mL). CHAP_k_ reduced bacteria by 3.3 log_10_ CFU/mL within 25 min, whereas that of vancomycin was 0.9 log_10_ CFU/mL, which showed significant differences in bacteria kiling activity between CHAP_k_ and vancomycin groups (*p* < 0.001) (Figure 2a,b). Considering the potential use of CHAP_k_ as mastitis therapeutics, the antimicrobial activity of CHAP_k_ in milk was evaluated. CHAP_k_ also retained high activity in fresh milk, which caused 2.4 log_10_ CFU/mL reduction of S. agalactiae within 25 min, however, vancomycin showed only 0.9 log_10_ CFU/mL reduction.

### 3.4. SEM Observation 

**Figure 3:**
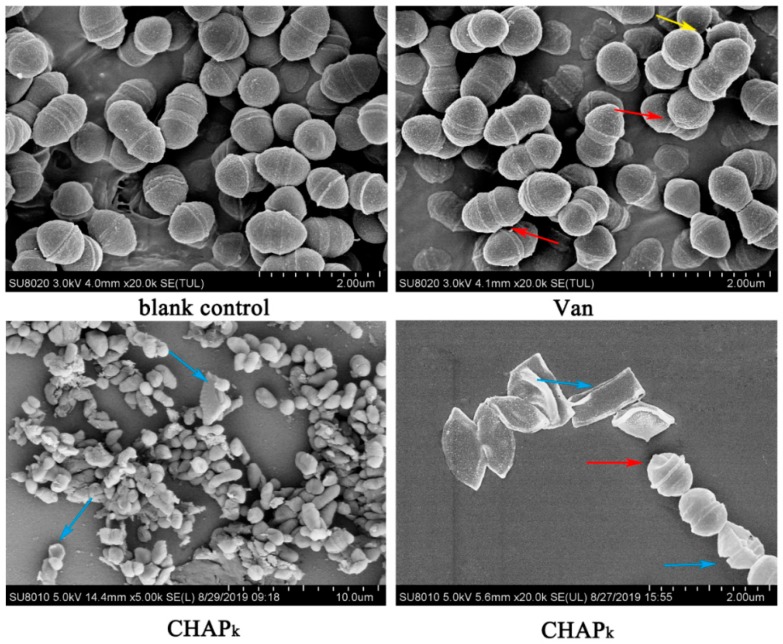
Scanning electron microscope observation. The magnification of the image is 20,000 (blank control), 20,000 (Van), 5000 (CHAP_k_-left bottom), and 20,000 (CHAP_k_-right bottom), respectively. Red arrows: Cell shrinkage; Yellow arrows: Vesicular bulge; Blue arrows: Cell rupture; Van: vancomycin.

The changes in cell morphology, integrity, and cellular structure of *S. agalactiae* ATCC 13813 were directly observed by SEM after treatment with CHAP_k_ or vancomycin. The untreated bacteria were ovoid and clustered together, displaying morphologic integrality, and the cell surface was smooth (Figure 3). After treatment with vancomycin, wrinkles on the cell surface were observed, some cells were slightly deformed, but most of the cells remained intact. However, after treatment with CHAP_k_, the number of bacteria was significantly reduced, cells wrinkled seriously and even ruptured, implying that CHAP_k_ and vancomycin exhibit different bactericidal mechanisms. 

### 3.5. Ability of CHAP_k_ against Biofilms and Bacteria of S. agalactiae

#### 3.5.1. Biofilms Formation Capacity of S. agalactiae ATCC 13813

According to the evaluation criteria of biofilms formation capacity based on crystal violet staining method, the absorbance value of *S. agalactiae* ATCC 13813 (0.361 ± 0.04) was four times higher than that of blank control (0.089 ± 0.01) at OD570 nm, which indicated that *S. agalactiae* ATCC 13813 is a strong biofilm-forming strain. 

#### 3.5.2. Effect of CHAP_k_ on S. agalactiae ATCC 13813 Early and Mature Biofilms

**Figure 4:**
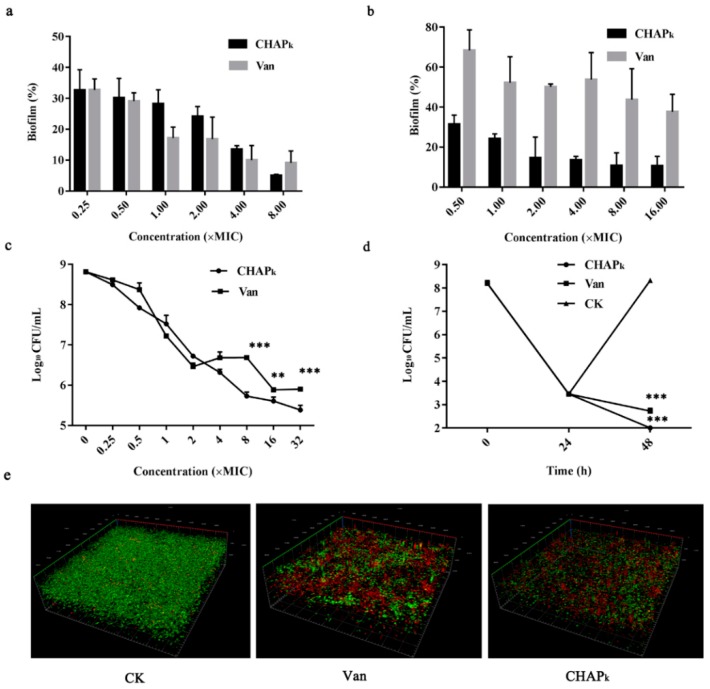
Effects of CHAP_k_ on *S. agalactiae* biofilms and bacteria in biofilms. (**a**) Inhibition of biofilms formation by CHAP_k_. (**b**) Eradication of mature biofilms by CHAP_k_. (**c**) Bactericidal activity against the early biofilms of *S. agalactiae*. (**d**) Bactericidal activity of CHAP_k_ against the mature biofilms of *S. agalactiae*. (**e**) Observation of *S. agalactiae* biofilms by CLSM. *S. agalactiae* was incubated with 8 × MIC CHAP_k_ or vancomycin for 24 h; after removing planktonic bacteria, biofilms were stained with dyes and visualized by CLSM. Live cells are stained in green by SYTO9 and dead cells are stained in red by PI, CK: the untreated *S. aureus* biofilms. Van: vancomycin. All assays were performed in triplicate. The analyses were measured by one-way ANOVA, with Duncan’s multiple comparisons test. A probability value of < 0.05 was considered significant. (*) Indicates the significance between control and treatment groups. ** *p* < 0.01; *** *p* < 0.001. The results are given as the mean ± SD (*n* = 3).

Early biofilm showed obvious signs of cell-cell aggregation. To investigate the inhibitory effect of CHAP_k_ on early biofilms, different concentrations of CHAP_k_ were exposed to *S. agalactiae*. After treatment with 0.25–8 × MIC CHAP_k_ or vancomycin, the biofilms were inhibited by 67.3–95% and 67.3–90.9%, respectively, whose inhibitory effect was displayed in a concentration-dependent manner (Figure 4a). These data suggested that CHAP_k_ has a potent inhibition ability to early biofilms, which is similar to vancomycin.

Peptidoglycan further bound and fully deployed to form mature biofilms. Mature biofilms are more difficult to remove than early biofilms. The eradication rate of mature biofilms was only 31% after exposed to 0.5 × MIC vancomycin, which was much lower than that of early biofilms. After treatment with 16 × MIC vancomycin, 62.3% mature biofilms were eradicated (Figure 4b). At the concentration of 0.5–16 × MIC CHAP_k_, the eradication rate of mature biofilms reached 68.4–89.4%, which was superior to that of vancomycin. CHAP_k_ eradicated the mature biofilms in a concentration-dependent manner. It demonstrated that CHAP_k_ has a stronger ability to eradicate the mature biofilms than vancomycin.

#### 3.5.3. Effect on Bacteria in Early and Mature Biofilms

The growth of bacteria in early biofilms was investigated by plate counting. *S. agalactiae* ATCC 13813 cells in early biofilms significantly declined after exposure to CHAP_k_ (Figure 4c). At the concentrations of 0.25–4 × MIC, there were no significant differences in bacterial reduction between CHAP_k_ and vancomycin, but at the concentrations of 8–32 × MIC, the bacterial reduction caused by CHAP_k_ was superior to that of vancomycin, and there were significant differences between CHAP_k_ and vancomycin groups (*p* < 0.01). The results indicated that CHAP_k_ can effectively kill bacteria in early biofilms of *S. agalactiae* in a concentration-dependent manner, which was in accordance with results of its inhibitory effect on biofilms in early stages. 

To further explore the activity of CHAP_k_ against bacteria in mature biofilms which were resistant to vancomycin, the mature biofilms of *S. agalactiae* ATCC 13813 were exposed to 25 × MIC vancomycin for 24 h, the bacteria encapsulated in biofilms reduced from 10^8^ CFU/mL to 10^3^ CFU/mL and no colonies were regrown. The bacteria in mature biofilms were significantly killed by 99% with 4 × MIC CHAP_k_ in 24 h, which was superior to vancomycin (Figure 4d). It indicated that CHAP_k_ has the potential activity to kill the vancomycin-resistant bacteria.

#### 3.5.4. Observation of Biofilms by CLSM

To further confirm the inhibition and eradication effects of biofilms and internal bacteria, the *S. agalactiae* ATCC 13813 cells were treated with SYTO9 and PI, and observed by CLSM (Figure 4e), the thickness of biofilms formed by untreated *S. agalactiae* in confocal dish reached 20.72 μm. Compared with untreated group, the biofilms significantly became thinner (thickness of 11.28 μm) and dead bacteria increased in CHAP_k_ treatment group, which was superior to vancomycin treatment group, implying the strong activity of CHAP_k_ against *S. agalactiae* and the biofilms.

## 4. Discussion

*S. agalactiae* is a contagious pathogen which mainly causes bovine intramammary infections and spreads to the herd [34,35,36,37,38]. With the increase of antibiotics resistance, especially for β-lactam antibiotics [8], novel antimicrobial agents are urgently needed. More recently, the original application of phage as therapeutics to treat human and animal infections has been rekindled, lysins have attracted attention again due to their specific antimicrobial activity after being discovered for a century [12,39]. Deeper research is now involved in lysins, especially for their effect on Gram-positive bacteria. CHAP_k_ is reported truncated phage lysin and it presents a broader antimicrobial spectrum as same as the original LysK enzyme. Compared with native enzyme LysK, the truncated single-domain lysin, CHAP_k_, showed the same lytic activity [22]. CHAP_k_ displays strong inhibition activity against *S. aureus*, including methicillin resistant *S. aureus* (MRSA) and vancomycin-intermediate *S. aureus* (VISA) strains [19,22,23,24,25,26]. CHAP_k_ also has some antibacterial effects against streptococcus [21]. In this study, it was firstly demonstrated that CHAP_k_ had the anti-biofilm and inhibition of bacteria in biofilms effects on *S. agalactiae.* In this study, CHAP_k_ has potent antibacterial activity against *S. aureus*, the MIC values were 0.43–3.44 μM (Table 1), this result is consistent with the conclusions of former research in this field, which demonstrated the antimicrobial activity against *S. aureus* in vitro [19,21,22,25,40]. Meanwhile, CHAP_k_ also displayed antimicrobial activity for *S. agalactiae* isolated from bovine mastitis. Previous studies have demonstrated that CHAP_k_ can rapidly lyse *S. aureus*, for *Streptococcus* (*S. mutans* DSM 6178 and *S. pneumoniae* DSM 11865), small decrease in turbidity was observed after treatment with CHAP_k_, which means less cell death [26], but little research in this field about *S. agalactiae*. In this study, the antimicrobial activity of CHAP_k_ has been investigated against *S. agalactiae*. CHAP_k_ exhibited high antibacterial activity against *S. agalactiae* (MIC: 0.05–0.11 μM), and also displayed a potent effect against the other streptococcus, including *S. uberis* and *S. dysgalactiae*, which were also the pathogens causing bovine mastitis. These results suggest that CHAP_k_ may work at a common part in the peptidoglycan cross-bridge of staphylococcal and streptococcal pathogens. CHAP_k_ cleaves the staphylococcal cell wall on the peptide bond between D-alanine and the first glycine in the pentaglycine cross bridge [19,26], whereas the streptococcal peptidoglycan cross-bridge contains a D-alanine-L-alanine bond and no glycine residues [41]. It was reported that the Cpl-7 cell wall binding domains of the streptococcal phage lysin λSa2 were replaced by staphylococcal SH3b domains from phage lysin LysK, which resulted in increased staphylolytic activity by five times based on the maintained streptolytic activity, suggesting that the staphylococcal domains have certain broad-spectrum antibacterial properties that are not always staphylococcal-specific [42]. The activity of CHAP_k_ against *S. agalactiae* is worth further investigation. In this study, time-killing kinetic curves showed that CHAP_k_ had a rapid effect on bacteria both in TSB medium and in milk within 25 min, which was superior to vancomycin (Figure 2), and the result was accordance with the former research that CHAP_k_ retained antimicrobial activity in raw bovine milk [40]. However, the effect of CHAP_k_ was weaker in milk than that in TSB medium, this might include reduced affinity to the *S. agalactiae* cell envelope which was changed when grown in milk or CHAP_k_ bound to milk components [43]. Because of the special mode of inhibition, lysins have no effect on commensal microflora, which is also a reason for the safety of using CHAP_k_ [44,45,46]. In addition, the destructed cell wall and leaked contents indicated that CHAP_k_ kills *S. agalactiae* by causing cells to rupture (Figure 3). The activity of CHAP_k_ truncated cell-wall binding domain (CBD) has been suggested to be connected with the overall charge of the enzymatically active domain (EAD) alone, seemingly because EAD with positive charge can independently bind the bacterial cell wall of the CBD [44].

Biofilms provide a habitat or reservoir for bacteria, which make it difficult for antibiotics to work, leading to resistances and reinfections [47,48]. A previous study has demonstrated that CHAP_k_ has the anti-biofilm properties for *S. aureus* [41]. In this study, CHAP_k_ has the effect on early biofilms (24 h growth) and mature biofilms (48 h growth) in a concentration-dependent manner (Figure 4a,b) [49]. For the early biofilms, CHAP_k_ had potent inhibited ability which is similar to vancomycin, the reason may be that in the early stage of biofilms production, low yield of biofilms is insufficient to provide protection for bacteria, antibiotics can inhibit the formation of biofilms by killing the *S. agalactiae*. However, for mature biofilms, CHAP_k_ displayed higher eradication rate than that of vancomycin, and previous research indicated there was no changes in other streptococcus (*Streptococcus anginosus*) biofilms by vancomycin exposure [50]. The results demonstrated that CHAP_k_ had a more potent anti-biofilm ability than vancomycin, which is consistent with the observation of biofilms by CLSM (Figure 4e). CHAP_k_ also successfully disrupted the *S. aureus* biofilms at a concentration as low as 3.91 μg/mL, there was little or no visible biofilms detected at a concentration of 62.5 μg/mL [51]. The antibiofilm activity of chimeric phage endolysin Ply187 on biofilms of MRSA strains were measured by live/dead staining assay, which indicated effective antimicrobial activity, whereas gentamycin had a poor effect on biofilms viability [48], which indicated that the lysins have better anti-biofilm activity than antibiotics. The bacteria in biofilms is a major reason that causes reinfections. CHAP_k_ can effectively reduce the number of *S. agalactiae* ATCC 13813 in biofilms, which showed a significant difference with vancomycin. Earlier studies have shown that vancomycin that inhibit cell wall synthesis reduce bacterial adherence, thus reducing bacteria and biofilm formation [52]. Recent research showed that the resistance of *Streptococcus anginosus* to vancomycin in a multispecies biofilm is due to increased thickness of the cell wall [50]. However, the elimination of biofilms by lysin is in connection with lysin inhibiting poly-intercellular adhesion (PIA) binding to peptidoglycan by rapidly degrading the cell wall and significantly decreasing the eDNA content. This may be the reason why CHAP_k_ is more potent in killing *S. agalactiae* and inhibiting biofilms formation than vancomycin [53]. The bacteria in biofilms show reduced susceptibility to antibiotics among several important pathogens [54], in contrast, lysins have high efficiency activity against bacteria in biofilms. CHAP_k_ killed the bacteria in biofilms, which were resistant to 25 × MIC vancomycin. Survival of bacteria in *S. aureus* biofilms were formed by treatment with 2 mg/mL of rifampicin or 3 mg/mL of ciprofloxacin for 4 h, which could be eliminated with 0.5 mM endolysin LysH5 [55]. After treatment with 1 × MIC of P128, the survival of *S. epidermidis* in 50 × MIC daptomycin or 100 × MIC vancomycin could be drastically reduced [56].

In summary, recombinant CHAP_k_ was successfully expressed in *E. coli* via pET28a, CHAP_k_ exhibited potent antimicrobial activity against Gram-positive, especially for *S. agalactiae* which were isolated from bovine mastitis. CHAP_k_ could rapidly lyse *S. agalactiae* cells in a short time, and it also significantly inhibited its biofilms formation at an early stage and eliminated its mature biofilms. For the bacteria in biofilms, CHAP_k_ showed efficient antimicrobial ability, which was superior to vancomycin. The potent antimicrobial activity of CHAP_k_ against the *S. agalactiae* isolated from bovine mastitis is the basis for its clinical application. It is suggested that CHAP_k_ may be a candidate for novel antimicrobial agents against streptococcal and even staphylococcal infections in mastitis treatment.

## Figures and Tables

**Table 1:** The MIC values of CHAP_k_ and vancomycin.

Strain			MIC	
	CHAP_k_		Vancomycin	
	μg/mL	μM	μg/mL	μM
Gram-positive bacteria				
*Streptococcus agalactiae* ATCC 13813	2	0.11	1	0.67
*S. agalactiae* CAU-FRI 1	1	0.05	1	0.67
*S. agalactiae* CAU-FRI 2	1	0.05	1	0.67
*S. agalactiae* CAU-FRI 3	1	0.05	1	0.67
*S. agalactiae* CAU-FRI 4	2	0.11	1	0.67
*Streptococcus uberis* CAU-FRI 1	2	0.11	0.5	0.34
*S. uberis* CAU-FRI 2	0.5	0.03	0.5	0.34
*S. uberis* CAU-FRI 3	1	0.05	0.5	3.34
*Streptococcus dysgalactiae* CAU-FRI 1	4	0.22	0.5	3.34
*S. dysgalactiae* CAU-FRI 2	2	0.11	1	0.67
*S. dysgalactiae* CAU-FRI 3	4	0.22	0.5	0.34
*Streptococcus pneumoniae* CVCC 3309	1	0.05	1	0.67
*S. pneumoniae* CVCC 2350	8	0.43	4	2.69
*S. pneumoniae* CGMCC 1.8747	16	0.86	4	2.69
*Streptococcus suis* CVCC 606	2	0.11	1	0.67
*S. suis* CVCC 3928	2	0.11	1	0.67
*Staphylococcus aureus* KY	8	0.43	4	2.69
*S. aureus* KR	8	0.43	4	2.69
*S. aureus* FJ	32	1.72	8	5.38
MRSA *S. aureus* ATCC 43300	64	3.44	8	5.38
*S. aureus* CVCC 546	64	3.44	8	5.38
*Staphylococcus hyicus* NCTC 10350	32	1.72	4	2.69
*Staphylococcus epidermidis* ATCC 12228	32	1.72	4	2.69
*S. epidermidis* ATCC 35984	16	0.86	4	2.69
Gram-negtive bacteria				
*Escherichia coli* ATCC 25922	>128	>6.88	>128	86.14
*Salmonella enteritidis* CVCC 3377	>128	>6.88	>128	86.14
Fungi				
*Candida albicans* CGMCC 2.2411	>128	>6.88	>128	86.14
